# Effects of two ventilator-weaning methods on lung volume and ventilation distribution by electrical impedance tomography in post-cardiac surgery patients: a prospective cohort study

**DOI:** 10.1186/s40560-026-00850-1

**Published:** 2026-01-23

**Authors:** Song Zhang, Siyi Yuan, Songlin Wu, Yi Chi, Haoping Huang, Shulin Zhang, Yingying Yang, Qianlin Wang, Fang Wang, Longxiang Su, Zhanqi Zhao, Huaiwu He, Yun Long

**Affiliations:** 1 State Key Laboratory of Complex Severe and Rare Diseases, Department of Critical Care Medicine, Peking Union Medical College Hospital, Peking Union Medical College, Chinese Academy of Medical Sciences, Beijing, China; 2 Department of Critical Care Medicine, The Affiliated Hospital, Southwest Medical University, Luzhou, Sichuan China; 3 School of Biomedical Engineering, Guangzhou Medical University, Guangzhou, China

**Keywords:** Electrical impedance tomography, Spontaneous breathing trial, End-expiratory lung volume

## Abstract

**Background:**

The effect of different spontaneous breathing trial (SBT) methods on lung volume and ventilation distribution has not been well clarified in post-cardiac surgery patients.

**Methods:**

In this prospective observational study, patients underwent 30 min of pressure-support ventilation (PSV)-SBT [PS 8 cmH_2_O, zero positive end-expiratory pressure (ZEEP)], followed by a 30-min T-piece trial if tolerated. Electrical impedance tomography (EIT) was used to continuously monitor regional lung ventilation and end-expiratory lung volume (EELV) at baseline, PSV-SBT 3 min, PSV-SBT 30 min, T-piece SBT 3 min and T-piece SBT 30 min. EELV_loss_ = [VT_baseline_/tidal impedance variation (TIV)_baseline_] × ΔEELI. EELV_loss PSV_ was defined as volume loss at 30 min of PSV-SBT and EELV_loss T-piece_ was defined as volume loss during T-piece SBT.

**Results:**

In 60 patients who complied with both SBT steps, 43 succeeded (71.7%) and 17 failed (28.3%) the T-piece SBT. Compared to the success group, the failure group exhibited a higher incidence of pendelluft (52.9% vs. 23.3%, *p* = 0.045) and significantly greater EELV_loss_ at T-piece SBT 30 min (623[459,746] ml vs. 511[376,702]ml, *p* = 0.003). However, the success group showed greater EELV_loss PSV_ than the failure group (322[247,459] ml vs. 199[166, 269] ml, *p* < 0.001), which was an abnormal pattern. Notably, the failure group had lower TIV (2102[1769,2562] vs. 2742[2153,3872], *p* = 0.005) and a higher respiratory rate (RR) than baseline at PSV-SBT 30 min (20[17,24] vs. 16[12,18], *p* < 0.001). Furthermore, we classified all patients into two groups based on the predominant reduction of volume loss: *P*-volume loss group (*N* = 37, EELV_loss PSV_ > EELV_loss T-piece_) and T-volume loss group (*N* = 23, EELV_loss T-piece_ > EELV_loss PSV_). In addition, the T-volume loss group had a higher weaning failure rate than the P-volume loss group (52.2% [12/23] vs. 13.5% [5/37], *p* < 0.001) and was associated with reduced baseline dorsal ventilation (39%[37%,43%] vs. 44%[41%,50%], *p* = 0.023). ROC analysis suggested that a dorsal ventilation threshold of 40.5% was associated with *T*-volume loss.

**Conclusions:**

The successful weaning patients had a higher reduction of EELV_loss PSV_ and a lower reduction of EELV_loss T-piece_. In the weaning failure patients, the paradox of lower EELV_loss PSV_ that was accompanied by a high RR and low VT might be associated with air trapping. Attention should be paid to using EELV_loss PSV_ to identify weaning failure.

**Supplementary Information:**

The online version contains supplementary material available at 10.1186/s40560-026-00850-1.

## Introduction

Spontaneous breathing trials (SBTs) are a critical component in deciding whether to extubate. Approximately 15%–20% of patients extubated following planned weaning require reintubation, which significantly prolongs hospital stays and worsens patient outcomes [1–4]. Therefore, it is crucial to select an appropriate SBT approach in patients at high risk of extubation failure. The most common SBT in North America is pressure-support ventilation (PSV) with positive end-expiratory pressure (PEEP), whereas, in Europe, SBTs are often performed with a T-piece or with low levels of pressure support without PEEP [5, 6].

The less challenging PSV-SBT is easier to pass but perhaps cannot truly evaluate the patient’s capacity to maintain inspiratory effort. At the same time, T-piece SBT may prolong the duration of mechanical ventilation [7, 8]. Although prior studies suggested that PSV-SBT may hasten extubation compared to T-piece SBT and without increased risk of reintubation, most of these patients were at low risk of extubation failure and the findings might not be extrapolable to patients at high risk [9, 10]. The underlying respiratory physiological effects of these different SBT methods still remain insufficiently explored.

Electrical impedance tomography (EIT) is a bedside, non-invasive monitoring technique that provides real-time information on regional tidal ventilation and lung volume changes [11, 12]. It has certain predictive value for the weaning results of patients on mechanical ventilation [13, 14]. EIT can also detect pendelluft, a phenomenon where intrapulmonary gas shifts between dependent and non-dependent lung regions, and this has been associated with SBT failure [15].

End-expiratory lung impedance (EELI) is well correlated with end-expiratory lung volume (EELV), which probably reveals alveolar recruitment and derecruitment and seems to play a key role in the evaluation of weaning outcome [16]. A previous study has shown that PSV-SBT and T-piece SBT both had the lung volume reduction, and the decreased lung volume of the T-piece was greater (approximately 800 ml) when compared with the PSV (approximately 400 ml) [17], but the correlation of decreased EELV and SBT failure in critically ill patients is not well defined. This study attempted to investigate the effect of EIT parameters on weaning outcomes between different SBT methods, reflect the physiological characteristics of different SBT methods in patients at high risk of extubation failure, and explore the relationship of volume loss and SBT failure.

## Methods

### Patient population

This study is a prospective observational study that enrolled patients in the intensive care unit of Peking Union Medical College Hospital from July 2024 to November 2024. We included patients older than 18 years who were mechanically ventilated for at least 24 h and attempted weaning for the first time after cardiac surgery and were considered at high risk of extubation failure (i.e., age > 65 years, or P/F < 300 mmHg, or with underlying chronic heart disease or chronic respiratory disease).

In addition, patients had to meet the criteria for weaning before starting an SBT [2]: respiratory rate ≤ 35 breaths per minute, peripheral oxygen saturation (SpO_2_) > 90% with FiO_2_ ≤ 0.4 or P/F ≥ 150 mmHg with PEEP ≤ 8 cmH_2_O, adequate cough, Glasgow Coma Scale score > 13 or Richmond Agitation–Sedation Scale score between − 1 and 1, and low dosages of vasopressors. Patients with contraindications for EIT (pregnancy, chest wound, chest deformity, pacemakers, etc.) were excluded from the study.

This study was in accordance with the Declaration of Helsinki and approved by the Ethics Committee for clinical studies of the Peking Union Medical College Hospital (K6371). The informed consent was obtained from all patients or next of kin before enrollment.

### Study design and monitoring protocol

After enrollment under adequate sedation and analgesia, patients remained on PSV with baseline settings of PS 8 cmH_2_O and PEEP 5 cmH_2_O, titrated to SpO₂ 95–98% with a fixed FiO_2_. PEEP was then abruptly reduced to 0 cmH_2_O while PS was kept at 8 cmH_2_O; this first PSV-SBT lasted 30 min. Patients who tolerated it proceeded immediately to a 30-min T-piece trial. The trial was terminated at any point if predefined SBT failure criteria appeared: respiratory rate (RR) ≥ 35 breaths min^−1^ or ≥ 50% increase from baseline, accessory muscle use, SpO_2_ < 90%, PaO_2_ ≤ 50 mmHg on FiO_2_ ≥ 0.5, altered consciousness, diaphoresis, or overt respiratory distress; they were then returned to the pre-SBT PSV settings.

At the end of T-piece SBT, the endotracheal tube would then be removed if patients met the extubation criteria and the extubation decision was assessed by two independent clinical attending physicians. Extubation criteria included SpO_2_ > 95%, RR < 30 breaths min^−1^, hemodynamically stable, presence of cough reflex, and the ability to protect the airway. Reintubation was performed if the patients presented difficulties in breathing and could not be alleviated by non-invasive positive pressure ventilation.

EIT and physiologic data were captured at five defined time-points:

1. Baseline before SBT start;
2. 3 min after PEEP was set to 0 cmH₂O (PSV-SBT 3 min);
3. Shortly before the end of 30-min PSV-SBT (PSV-SBT 30 min);
4. 3 min into the T-piece trial (T-piece 3 min);
5. Before the end of the 30-min T-piece trial (T-piece 30 min).

At each time-point we recorded regional ventilation by EIT (PulmoVista500, Dräger Medical, Germany; 20 Hz sampling rate) via a 16-electrode silicone belt positioned at the 4th intercostal space while the patient lay supine with 30° head-up tilt, together with simultaneous vital signs (blood pressure, heart rate, respiratory rate, SpO_2_) and baseline respiratory mechanics.

### EIT data analysis

EIT data analysis was performed offline using dedicated software (Draeger EIT Data Analysis Tool Ver 6.3; Dräger Medical, Germany). For the analysis of regional ventilation, the EIT image was divided into four regions of interest (ROIs), from ventral to dorsal, each covering 25% of the ventro-dorsal diameter (ROI 1–4) [18–20]. EELI was averaged over six breaths for the whole lung and was defined as the local minimum of the global impedance curve [16].

- EELV_loss_ = (VT_baseline_/TIV_baseline_) × ΔEELI [21].
- EELV_loss PSV_ = EELV_PSV-SBT30min_ − EELV_PSV-SBT3min_.
- EELV_loss T-piece_ = EELV_T-piece30min_ − EELV_T-piece3min_.

Comparison of the two volume losses classified into two groups:

- P-volume loss: EELV_loss PSV_ > EELV_loss T-piece_ (volume loss mainly under PSV).
- T-volume loss: EELV_loss T-piece_ > EELV_loss PSV_ (volume loss mainly under T-piece).

The global inhomogeneity index (GI) and center of ventilation (CoV) were calculated to indicate ventilation distribution inhomogeneity [22, 23]. The regional ventilation delay (RVD) index was used to describe the delay between the beginning of inspiration and the culmination of a specific impedance threshold and may correlate with tidal recruitment within the lung [24]. The EIT-based pendelluft amplitude was defined as the impedance difference between the sum of all regional TIV and the global TIV [25]. The occurrence of pendelluft was considered when its amplitude exceeded 3% of global TIV [15].

### Statistical analysis

We assumed a SBT failure rate of 25%–40% for patients at high risk of extubation failure [26]. The primary physiological endpoint of this study was the between-group difference in ΔEELV (ml) at the T-piece SBT. Based on our pilot data (*n* = 10 per group), the mean ΔEELV was 240 ml (SD 400 ml), yielding an expected effect size *d* = 0.60. With a significance level (alpha) set at 0.05 and a desired power (1-beta) of 0.80, the sample size calculation determined that a minimum of 60 patients would be required to demonstrate a significant difference in EELV_loss_ between SBT methods and outcomes, which could ensure the study has adequate statistical power to draw meaningful conclusions.

Data were analyzed using descriptive statistics to summarize the demographic and baseline characteristics of the study population. The normality of data was assessed using the Shapiro–Wilk test. The continuous variables with normal distribution were expressed as mean ± standard deviation (*x* ± *s*), while non-normally distributed continuous variables were presented as the median and interquartile range (25th–75th percentiles). The two independent-sample t test or Mann–Whitney *U* test were used to compare the differences between groups depending on the data distribution in continuous variables. Categorical variables were represented as numbers and proportions and were compared using Chi-square or Fisher’s precision probability test, as appropriate.

We used the linear mixed-effect (LME) model to estimate the differences in physiological data as well as EIT parameters from five time-points between the two groups. We modeled the independent variables (time and groups) as fixed effects, and a random-effect intercept for each participant was used in the model to account for the within-individual correlations. We used a multiple comparison correction method (Holm’s method) for family-wise error rate control and adjusted the *p* values. Logistic regression analysis was used to analyze the impact of volume-loss classification on the T-piece SBT failure. Sensitivity analyses (early vs late enrollees, stratification by total elapsed time, time as a covariate) were conducted to justify the fixed weaning sequence. A *p*-value < 0.05 was considered statistically significant. All statistical analyses were performed with SPSS 27.0 software package (SPSS, Chicago, IL), R (V.4.5.1) and GraphPad Prism 10.1.0.

## Results

### Patient characteristics

**Fig. 1:**
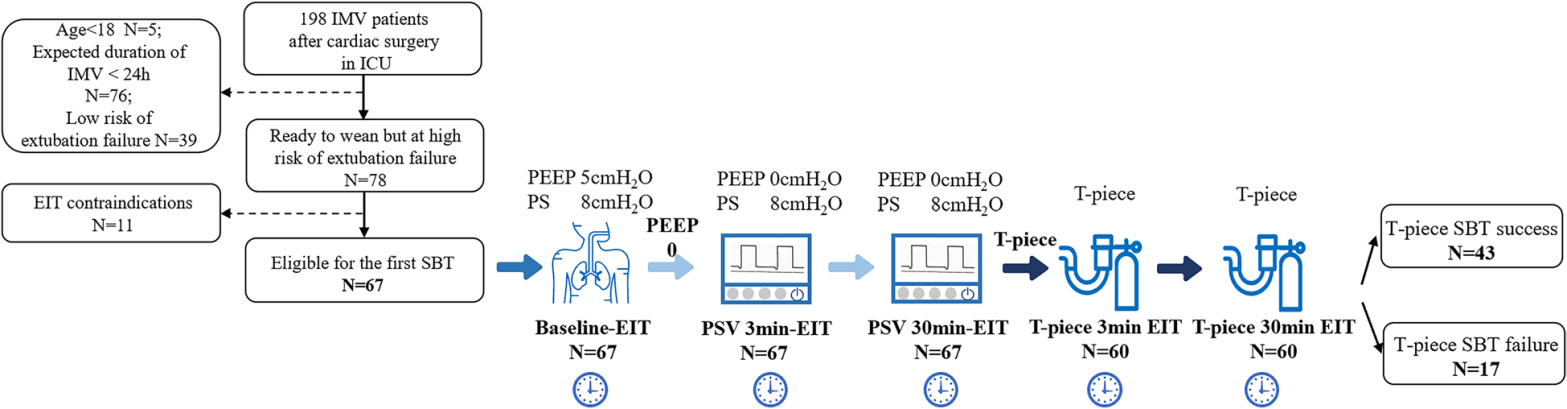
Flow diagram of the weaning process and patients’ inclusion details. SBT, spontaneous breathing trial; EIT, electrical impedance tomography; IMV, invasive mechanical ventilation; PSV, pressure-support ventilation; PS, pressure support; PEEP, positive end-expiratory pressure; ICU, intensive care unit

From July 2024 to November 2024, 198 patients receiving mechanical ventilation after undergoing cardiac surgery were admitted to the intensive care unit (ICU). Of these, 131 patients were excluded from the analysis. The causes of noninclusion are shown in the flowchart (Fig. 1). Finally, a total of 67 patients who were eligible for the first SBT were included and 7 patients (10.4%) failed the PSV-SBT. The baseline characteristics of these 7 most severely ill patients are shown in Supplementary Table 1.

Of the 60 patients who passed the PSV-SBT, 43 patients succeeded with the following T-piece SBT and were analyzed in the SBT success group; 17 patients (28.3%) who failed with the T-piece SBT were analyzed in the SBT failure group. The baseline patient characteristics and clinical outcomes are listed in Table 1. Patients were mostly male (55%) and the median age was 60 years (IQR, 49–67 years); the patients were older in the T-piece SBT failure group than the T-piece SBT success group (64 ± 13 vs. 56 ± 12, *p* = 0.036). The acute physiology and chronic health evaluation II (APACHE II) score on ICU admission day was higher for the patients who failed the T-piece SBT (14 ± 4 vs. 12 ± 4, *p* = 0.047). The type of surgery was not significantly different between the two groups (*p* > 0.05), but the surgery process was longer in the T-piece SBT failure group (CPB: 155 ± 41 vs. 125 ± 33, *p* = 0.012; AOC: 106 ± 30 vs. 82 ± 32, *p* = 0.024). The vital signs did not significantly differ between groups at the baseline (*p* > 0.05). There were no patients to extubate within 24 h of the T-piece SBT failure group and the rate of extubation within 24 h in the T-piece SBT success group was 88.4%. The T-piece SBT failure group had longer IMV days (7 [6, 9] vs. 5 [4, 6], *p* < 0.001) and lower duration of VFD at day 28 (21 [20, 22] vs. 23 [22, 24], *p* < 0.001) than those who succeeded in the T-piece SBT.

**Table 1:** Baseline clinical characteristics and demographics of patients

Variables	T-piece SBT success *N* = 43	T-piece SBT failure *N* = 17	*P*
Age, years	56 ± 12	64 ± 13	**0.036**
Sex, M (%)	25 (58.1)	8 (47.1)	0.44
BMI, kg/m^2^	24.4 ± 3.1	23.4 ± 3.2	0.27
APACHE II Score	12 ± 4	14 ± 4	**0.047**
Chronic respiratory disease, *n* (%)	6 (14.0)	5 (29.4)	0.15
Pre-operative EF value	63 [56,67]	64 [55,66]	0.83
Type of surgery			
CABG, *n* (%)	13 (30.2)	6 (35.3)	0.76
Valve surgery, *n* (%)	14 (32.6)	6 (35.3)	0.53
Others, *n* (%)	16 (37.2)	5 (29.4)	0.77
Surgery process			
CPB time	125 ± 33	155 ± 41	**0.012**
AOC time	82 ± 32	106 ± 30	**0.024**
P/F, mmHg	351 [290, 410]	318 [234, 462]	0.16
HR, bpm	90 [84, 96]	90 [83,99]	0.78
MAP, mmHg	89 [81, 95]	86 [76, 94]	0.37
RR, min^−1^	15 [12, 18]	16 [13, 18]	0.38
SpO_2_, %	99 [98, 100]	99 [98, 100]	0.35
NE dose, ug/kg/min	0.01 [0.00, 0.11]	0.00 [0.00, 0.15]	0.66
Extubation within 24 h, *n* (%)	38 (88.4)	0 (0.0)	< 0.001
IMV days in total^a^	5 [4, 6]	7 [6, 9]	< 0.001
VFDs at day 28	23 [22, 24]	21 [20, 22]	< 0.001
ICU stay, days	7 [6, 8]	7 [6, 9]	0.06
Reintubation more than 24 h, *n* (%)	0 (0.0)	0 (0.0)	/

Continuous variables are expressed as mean ± SD or median (25th–75th percentile), categorical variables are represented as values (%)

BMI, body mass index; APACHE, acute physiology and chronic health evaluation; EF, ejection fraction; CABG, coronary artery bypass grafting; CPB, cardiopulmonary bypass; AOC, aortic cross-clamping; P/F, arterial partial pressure of oxygen to inspired fraction of oxygen ratio; HR, heart rate; MAP, mean arterial pressure; RR, respiratory rate; NE, norepinephrine; SpO_2_, peripheral oxygen saturation; IMV, invasive mechanical ventilation; VFD, ventilator-free day; ICU, intensive care unit

^a^represents the period from the admission in ICU with mechanical ventilation to completely discontinuing mechanical ventilation, including the duration of reinitiating mechanical ventilation after SBT failure

Boldface indicates significant *P* values

### EIT variables throughout a two-step SBT sequence

EIT parameters during the two-step SBT sequence in T-piece SBT success and failure groups are displayed, respectively, in Table 2. Compared to the SBT success group, the SBT failure group showed a higher incidence of pendelluft at 30 min of the T-piece SBT (52.9% vs. 23.3%, failures vs. successes, *p* = 0.045). The other variables, including ventilation distribution, global inhomogeneity index, were not different after considering the interaction of time-points and groups (*p* > 0.05).

**Table 2:** The EIT variables during the weaning process

Variables	Time	Success group *N* = 43	Failure group *N* = 17	Group effect		Time effect			Group x time effect		Mean difference [95% CI]	P*
				*F*	*P*	*F*		*P*	*F*	*P*		
ROI 1 + 2 (%)	Baseline	55 [52,57]	57 [54,60]	0.158	0.69	5.794	< 0.001		0.115	0.95	− 0.507 [− 6.019, 5.006]	1.00
	PSV 3 min	53 [50,55]	56 [53,59]								− 1.915 [− 7.427, 3.597]	1.00
	PSV 30 min	54 [51,56]	55 [52,57]								− 0.834 [− 5.347, 4.124]	1.00
	T-piece 3 min	52 [49,57]	54 [48,58]								− 0.270 [− 5.782, 5.243]	1.00
	T-piece 30 min	52 [50,55]	55 [51,59]								− 0.541 [− 6.053, 4.971]	1.00
ROI 3 + 4 (%)	Baseline	45 [43,48]	43 [40,46]	0.158	0.69	5.794	< 0.001		0.115	0.95	0.507 [− 5.006, 6.019]	1.00
	PSV 3 min	47 [45,50]	44 [41,47]								1.915 [− 3.597, 7.427]	1.00
	PSV 30 min	46 [44,49]	45 [43,48]								0.834 [− 4.124, 5.347]	1.00
	T-piece 3 min	48 [43,51]	46 [42,52]								0.270 [− 5.243, 5.782]	1.00
	T-piece 30 min	48 [45,50]	45 [41,49]								0.541 [− 4.971, 6.053]	1.00
CoV	Baseline	49.4[47.9,52.0]	48.2[46.9,50.1]	1.021	0.99	0.632	0.60		0.481	0.70	3.491[− 13.781,20.764]	1.00
	PSV 3 min	49.5[48.1,51.7]	48.7[47.4,50.6]								3.379[− 13.893,20.652]	1.00
	PSV 30 min	49.9[48.7,52.3]	49.3[48.1,51.7]								3.154[− 12.783,18.895]	1.00
	T-piece 3 min	50.4[48.6,51.8]	48.5[47.1,50.6]								2.295[− 14.977,19.567]	1.00
	T-piece 30 min	50.0[48.1,52.0]	49.1[47.1,53.3]								− 9.355[− 27.519,8.810]	1.00
GI	Baseline	0.34[0.32,0.36]	0.34 [0.33,0.37]	0.172	0.68	2.782	0.043		0.682	0.56	0.009 [− 0.011, 0.030]	1.00
	PSV 3 min	0.34[0.32,0.36]	0.34 [0.33,0.37]								0.002 [− 0.019, 0.022]	1.00
	PSV 30 min	0.35[0.33,0.37]	0.34 [0.32,0.36]								0.004 [− 0.015, 0.026]	1.00
	T-piece 3 min	0.34[0.32,0.36]	0.35 [0.33,0.37]								− 0.007 [− 0.028, 0.013]	1.00
	T-piece 30 min	0.35[0.34,0.37]	0.35 [0.33,0.39]								0.008 [− 0.014, 0.029]	1.00
RVD	Baseline	3.1[2.3,5.0]	4.1[3.6,4.7]	1.668	0.20	0.988	0.40		0.823	0.48	1.284 [− 0.125, 2.693]	0.37
	PSV 3 min	3.3[2.6,5.2]	4.2[3.3,5.0]								0.905 [− 0.504, 2.314]	0.83
	PSV 30 min	3.4[2.4,5.0]	4.2[3.3,5.4]								0.638 [− 0.823, 2.072]	1.00
	T-piece 3 min	4.1[2.7,5.4]	4.4[3.5,5.6]								0.156 [− 1.254, 1.565]	1.00
	T-piece 30 min	4.2[2.9,5.6]	4.2[3.3,5.7]								0.243 [− 1.225, 1.712]	1.00
Pendelluft (%)	Baseline	8/43 (18.6)	3/17 (17.6)	0.688	0.41	4.602	0.001		1.827	0.12	− 0.022[− 0.123, -0.078]	1.00
	PSV 3 min	8/43 (18.6)	4/17 (23.5)								0.020 [− 0.145, 0.185]	1.00
	PSV 30 min	9/43 (20.9)	5/17 (29.4)								0.066 [− 0.224, 0.356]	1.00
	T-piece 3 min	9/43 (20.9)	6/17 (35.3)								1.190 [− 0.222, 0.602]	1.00
	T-piece 30 min	10/43 (23.3)	9/17 (52.9)								0.542 [0.127, 0.957]	0.045

PSV, pressure-support ventilation; EIT, electrical impedance tomography; GI, global inhomogeneity; CoV, center of ventilation; ROI, regions of interest; RVD, regional ventilation delay; CI, confidence interval

*The *p*-values adjusted through Holm’s method

**Fig. 2:**
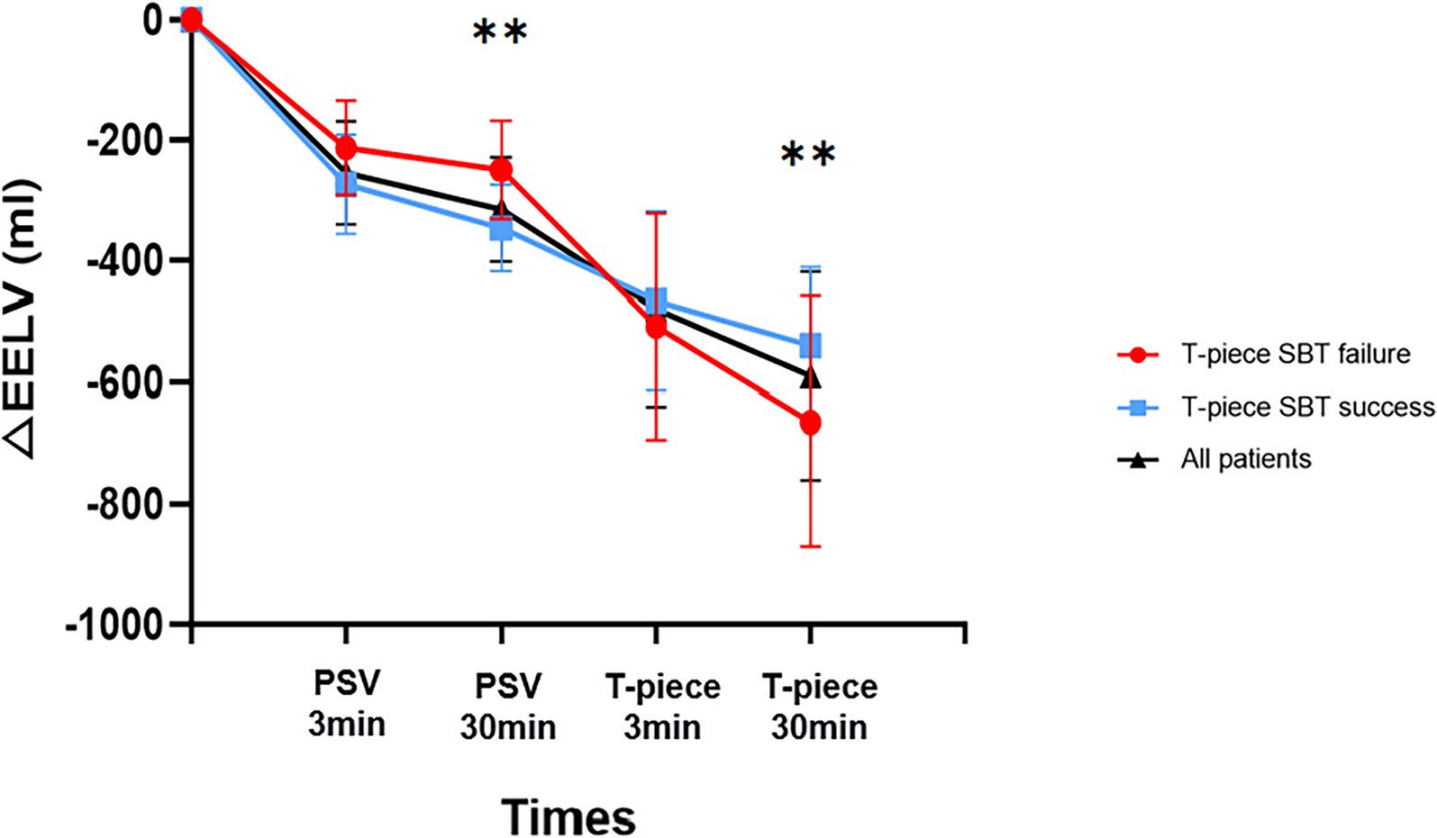
EELV_loss_ in all patients and different groups during the weaning process. EELV, end-expiratory lung volume; SBT, spontaneous breathing trial; PSV, pressure-support ventilation; **represents *p* < 0.01 between the T-piece SBT success group and T-piece SBT failure group

Figure 2 displays the changes of EELV_loss_ during the weaning process. The EELV_loss_ decreased gradually as the weaning process continued, but it did not decrease to the same extent at different time points in the T-piece SBT success and failure groups. The T-piece SBT success group had a greater reduction of EELV_loss_ compared with the T-piece SBT failure group at 30 min of PSV-SBT (322 [247, 459]ml vs. 199 [166, 269]ml, successes vs. failures, *p* = 0.006). The total EELV_loss_ was higher in the T-piece SBT failure group compared with the success group at the end of T-piece SBT (623 [459, 746]ml vs. 511 [376, 702]ml, failures vs. successes, *p* = 0.003). The SBT success group showed a greater reduction of EELV_loss_ at PSV-SBT than T-piece SBT (322 [247, 459]ml vs. 186 [129, 311]ml, PSV vs. T-piece, *p* < 0.001), while in the SBT failure group, EELV_loss_ was higher at T-piece SBT than PSV-SBT (400 [293, 436]ml vs. 199 [166, 269]ml, T-piece vs. PSV, *p* < 0.001). The results of the sensitivity analyses confirmed that time did not have any impact on the outcome of T-piece SBT failure and EELV_loss_. There was no significant difference in the rate of T-piece SBT failure among patients with different durations of ventilator time (IMV < 24 h, 2/7, 28.6%; 24 h-48 h, 4/15, 26.7%; > 48 h, 11/38, 28.9%, *p* = 0.98). There was also no significant difference of EELV_loss_ between the two periods (315[218,403] ml vs. 283[200, 389] ml, EELV_loss_ from baseline to PSV30min vs. EELV_loss_ from PSV30min to T-piece 30 min, *p* = 0.17). We also selected “time in protocol” as a covariate in the LME model and there was no difference (mean difference [95% CI] 1.854 [− 3.265, 5.732], *p* = 0.47).

**Fig. 3:**
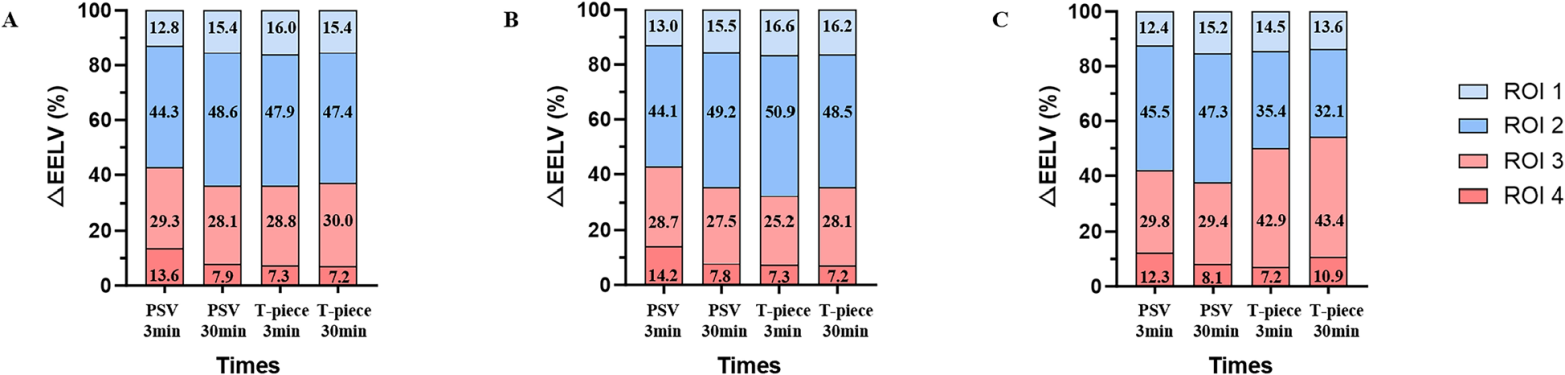
Changes of EELV_loss_ in different ROIs during the weaning process. **A** represents EELV_loss_ of different ROIs among all patients; **B** represents EELV_loss_ of different ROIs in T-piece SBT success group; **C** represents EELV_loss_ of different ROIs in T-piece SBT failure group; EELV, end-expiratory lung volume; ROI, regions of interest; SBT, spontaneous breathing trial; PSV, pressure-support ventilation

Regional analysis showed that EELV_loss_ during the T-piece SBT was spatially distinct between outcomes (Fig. 3). In patients who ultimately failed the T-piece SBT, the majority of the decrement was concentrated in the dependent ROI 3 (≈43% of total loss at 30 min; *p* = 0.017). Conversely, those who succeeded experienced a more homogeneous reduction, with the largest share occurring in the mid-ventral ROI 2 (≈49% of total loss; *p* = 0.024), and no preferential collapse in ROI 3.

**Fig. 4:**
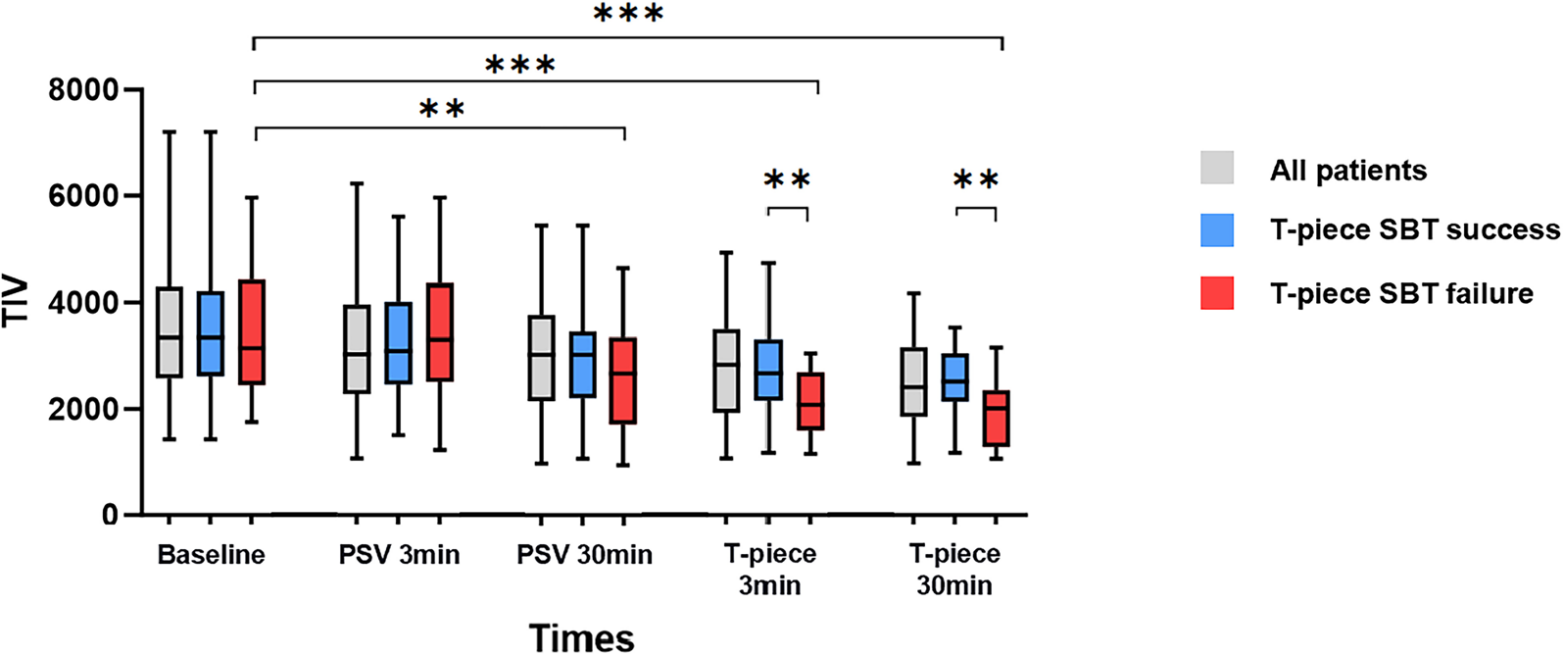
TIV in all patients and different groups during the weaning process. TIV, tidal impedance variation; SBT, spontaneous breathing trial; PSV, pressure-support ventilation; **represents *p* < 0.01; ***represents *p* < 0.001

In the failure group, TIV was lower at 30-min of PSV–SBT than the baseline (2102 [1769, 2562] vs. 2742 [2153, 3872], *p* = 0.005). At the same time, the SBT failure group had a lower value of TIV than the success group at both 3-min of T-piece SBT (2085 [1605, 2694] vs. 2679 [2161, 3315], failures vs. successes, *p* = 0.005) and 30-min of T-piece SBT (2018 [1287, 2355] vs. 2523 [2143, 3056], failures vs. successes, *p* = 0.003) in Fig. 4.

### Respiratory mechanical and hemodynamic parameters throughout a two-step SBT sequence

We also reported the vital signs at baseline, 3-min of PSV-SBT, 30-min of PSV-SBT, 3-min of T-piece SBT and 30-min of T-piece SBT in Table 3. The T-piece SBT failure group showed poorer RR and SpO_2_ than the success group during the T-piece SBT. In the failure group, RR was higher in 30-min of T-piece SBT than the baseline (24 [18, 27] vs. 16 [12, 18], *p* = 0.017). SpO_2_ (96 [95,98] vs. 100 [98,100], failures vs. successes, *p* = 0.004) was also significantly lower at 3-min of T-piece SBT in the T-piece SBT failure group compared to the success group, and the same trend was followed at 30-min of T-piece SBT. Heart rate (110 [94,117] vs. 91 [84,99], failures vs. successes, *p* = 0.034) was higher in the T-piece SBT failure group at the end of the weaning process.

**Table 3:** Respiratory mechanics variables and hemodynamic parameters during the weaning process

Variables	Time	Success group *N* = 43	Failure group *N* = 17	Group effect		Time effect		Group x time effect				Mean difference [95% CI]	*P*
				*F*	*P*	*F*	*P*	*F*			*P*		
RR,min^−1^	Baseline	15 [12, 17]	16 [12, 18]	4.472	0.039	3.101	0.017		1.736	0.14		4.180 [1.582, 6.778]	0.002
	PSV 3 min	16 [14, 18]	16 [12, 20]									0.689 [− 1.910, 3.287]	0.60
	PSV 30 min	16 [15, 19]	20 [17, 24]									2.194 [− 0.404, 4.792]	0.10
	T-piece 3 min	18 [15, 20]	22 [16, 27]									1.002 [− 1.596, 3.600]	0.45
	T-piece 30 min	18 [16, 20]	24 [18, 27]									0.461 [− 2.137, 3.060]	0.73
SpO_2_, %	Baseline	100 [98,100]	99 [98,100]	14.219	< 0.001	3.429	0.010		4.318	0.002		− 2.731 [− 3.874, − 1.588]	< 0.001
	PSV 3 min	100 [98,100]	98 [97,99]									− 0.134 [− 1.277, 1.008]	0.82
	PSV 30 min	100 [98,100]	97 [96,99]									− 1.193 [− 2.336, − 0.050]	0.041
	T-piece 3 min	100 [98,100]	96 [95,98]									− 1.691 [− 2.834, − 0.548]	0.004
	T-piece 30 min	100 [99,100]	96 [94,97]									− 1.909 [− 3.052, − 0.766]	0.001
HR, bpm	Baseline	90 [84,96]	90 [83,99]	0.078	0.78	7.396	< 0.001		4.654	0.001		− 5.563 [− 12.416, 1.290]	0.11
	PSV 3 min	91 [85,97]	95 [82,106]									0.291 [− 6.562, 7.144]	0.93
	PSV 30 min	91 [86,97]	99 [83,108]									2.294 [− 4.559, 9.147]	0.51
	T-piece 3 min	92 [85,99]	96 [81,109]									− 0.466 [− 7.319, 6.387]	0.89
	T-piece 30 min	91 [84,99]	110 [94,117]									7.436 [0.583, 14.289]	0.034
MAP, mmHg	Baseline	89 [85,94]	86 [76,94]	0.308	0.58	8.957	< 0.001		2.154	0.08		− 1.682 [− 7.767, 4.402]	0.58
	PSV 3 min	88 [84,93]	88 [79,95]									0.531 [− 5.554, 6.615]	0.86
	PSV 30 min	89 [83,96]	89 [81,96]									2.976 [− 3.108, 9.060]	0.33
	T-piece 3 min	91 [85,97]	91 [84,99]									1.362 [− 4.722, 7.446]	0.66
	T-piece 30 min	90 [87,96]	101 [90,113]									4.343 [− 1.741, 10.427]	0.16

PSV, pressure-support ventilation; RR, respiratory rate; SpO_2_, peripheral oxygen saturation; HR, heart rate; MAP, mean arterial pressure; CI, confidence interval

### *P*- and *T*-volume loss groups in relation to weaning outcome

**Fig. 5:**
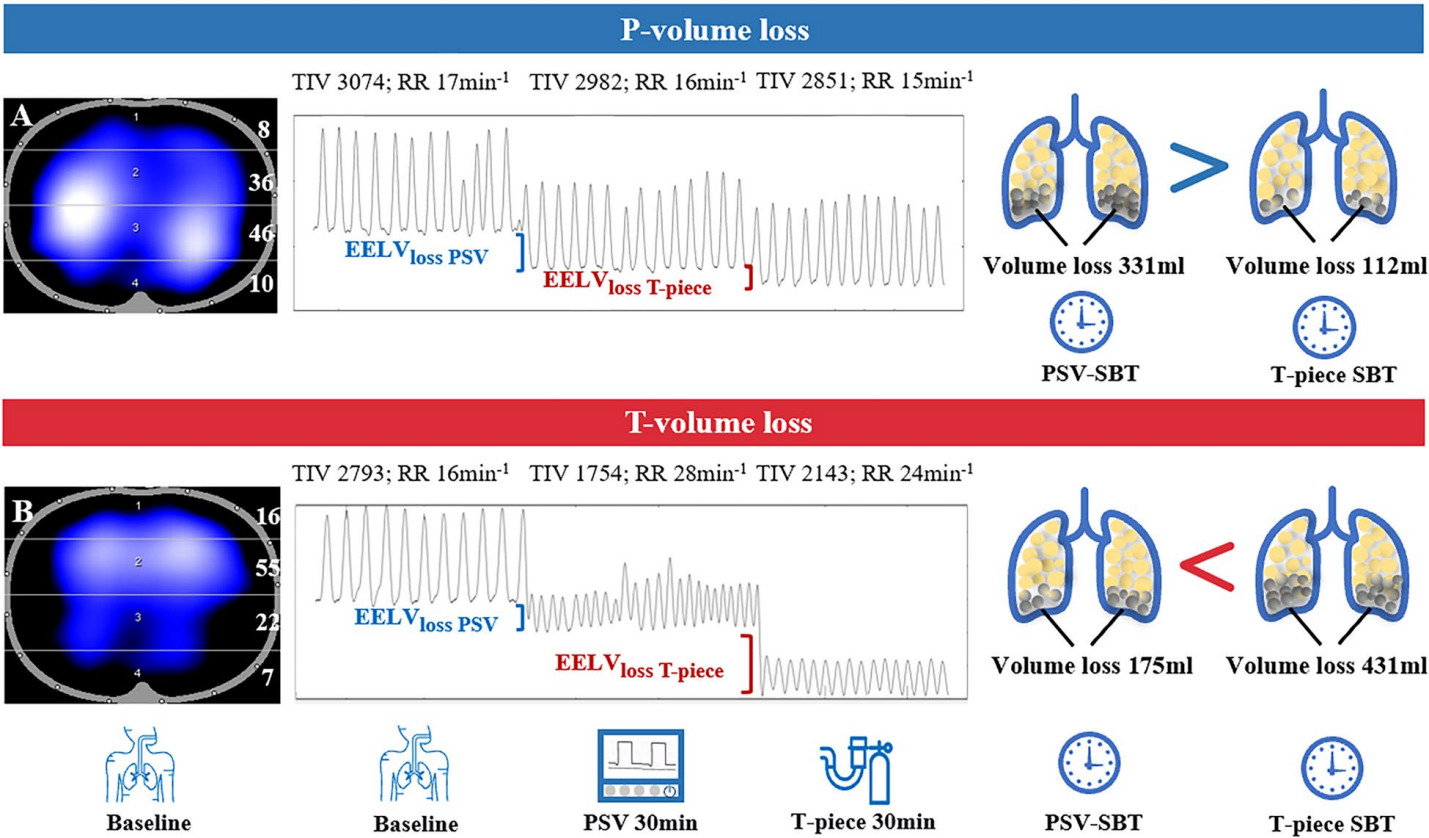
The schematic about two groups with the changes of EELV_loss_. *P*-volume loss represents EELV_loss PSV_ > EELV_loss T-piece_, *T*-volume loss represents EELV_loss T-piece_ > EELV_loss PSV_; **A** represents a patient with better dorsal ventilation (ROI 3 + 4: 56%) at baseline succeed the T-piece SBT and his lung volume loss occurred mainly at PSV-SBT; **B** represents a patient with worse dorsal ventilation (ROI 3 + 4: 29%) at baseline failed the T-piece SBT and his lung volume loss occurred mainly at T-piece SBT. EELV, end-expiratory lung volume; SBT, spontaneous breathing trial; PSV, pressure-support ventilation

Among the 60 patients who completed both SBT steps, the *T*-volume loss group (EELV_loss T-piece_ > EELV_loss PSV_) had a higher weaning failure rate (52.2% [12/23] vs. 13.5% [5/37], *p* < 0.001) than the *P*-volume loss group (EELV_loss PSV_ > EELV_loss T-piece_). Representative volume loss for each group is illustrated in Fig. 5. Multivariate logistic regression analysis showed that T-piece volume loss was independently associated with T-piece SBT failure (OR = 9.847, 95% CI 1.920–50.494, *p* = 0.006) in Supplementary Table 2. Baseline EIT characteristics diverged between groups (Table 4): patients assigned to the T-volume loss group already exhibited reduced dorsal ventilation (ROI 3 + 4: 39% [37%, 43%] vs. 44% [41%, 50%], *T*-volume loss vs. *P*-volume loss, *p* = 0.023) and an increase in ventral ventilation (ROI 1 + 2: 60% [55%, 64%] vs. 55% [51%, 59%], *T*-volume loss vs. *P*-volume loss, *p* = 0.014). We also illustrated that dorsal ventilation was independently associated with T-volume loss (OR = 0.805, 95% CI 0.686–0.946, *p* = 0.008) in Supplementary Table 3. At the same time, to evaluate the diagnostic value of baseline dorsal ventilation associated with T-volume loss classification, we drew ROC curve analysis and calculated the Youden index (Supplementary Fig. 1). The cutoff value for baseline dorsal ventilation determined by the highest Youden index was found to be 40.5%. The area under the curve was 0.787, with a sensitivity of 69.6%, specificity of 75.7%, and Youden index of 0.452. No significant differences were observed for global inhomogeneity, center of ventilation, regional ventilation delay, or prevalence of pendelluft (all *p* > 0.05).

**Table 4:** EIT variables of the two groups

EIT variables	Baseline		*P*
	*P*-volume loss *N* = 37	*T*-volume loss *N* = 23	
ROI 1 + 2 (%)	55[51,59]	60[55,64]	**0.014**
ROI 3 + 4 (%)	44[41,50]	39[37,43]	**0.023**
COV	49.4 [47.4,51.8]	47.9 [46.9,50.4]	0.12
GI	0.34 [0.33,0.36]	0.35 [0.33,0.37]	0.69
RVD	3.3[2.5,5.1]	4.2[3.7,4.6]	0.28
Pendelluft (%)	7/37 (18.9)	6/23 (26.1)	0.54

SBT, spontaneous breathing trial; EIT, electrical impedance tomography; GI, global inhomogeneity; COV, center of ventilation; ROI, regions of interest; RVD, regional ventilation delay

Boldface indicates significant *P* values.

## Discussion

This study is, to our knowledge, the first physiological exploration with EIT to map the entire step-down transition from PSV to T-piece SBT in post-cardiac surgery patients at high risk of extubation failure. We have depicted a physiological phenomenon about the lung ventilation during PSV-SBT to T-piece SBT and innovatively proposed the application misconceptions of EIT that the T-piece SBT success group showed greater EELV_loss PSV_ while the T-piece SBT failure group displayed lower EELV_loss PSV_. The abnormal pattern reminded us to be cautious to justly use EELV_loss PSV_ to identify weaning failure. Patients with worse dorsal ventilation (lower than 40.5%) may be more likely to fail the T-piece SBT, which is displayed as higher EELV_loss T-piece_. There were different physiological characteristics of EELV_loss_ during the sequential weaning process that represented an insightful view about weaning failure among critically ill post-cardiac surgery patients.

### Progressive EELV_loss_ and prediction of weaning failure

Throughout the weaning sequence, we observed a continuous decline in EELV_loss_. The magnitude of this loss, however, differed sharply between final outcomes. Patients who ultimately failed the T-piece SBT exhibited a significantly larger EELV_loss_ during the T-piece phase (EELV_loss T-piece_ = 400 mL) than during the preceding PSV phase (EELV_loss PSV_ = 199 mL). Conversely, those who succeeded showed the opposite pattern: the greatest volume loss occurred while PEEP was removed during PSV (EELV_loss PSV_ = 322 mL), whereas the subsequent T-piece trial added little further loss (EELV_loss T-piece_ = 186 mL).

EELV_loss PSV_ could not accurately reflect the occurrence of alveolar collapse or the SBT failure, which was an abnormal phenomenon. It suggests that the T-piece unmasks latent derecruitment that PSV-SBT—despite zero PEEP—may still conceal. Our research explained the physiological effects of different SBT methods under more stringent conditions as the transition from positive to negative pressure ventilation during the weaning process may induce cardiac dysfunction and lead to pulmonary edema. Inspiratory fall in intrathoracic pressure tends to increase the right ventricular preload, and the hypoxemia or worsening of intrinsic positive end-expiratory pressure may increase the right ventricular afterload in patients with pre-existing right ventricular disease [27, 28]. In the weaning failure patients, the paradoxical lower EELV_loss PSV_ accompanied by high RR and low VT might reflect alveolar derecruitment or hyperinflation. Air trapping might play a role in the reduction of EELI-based volume during PSV-SBT, but the accurate diagnosis of air trapping needs the measurements of esophageal pressure, expiratory flow limitation, intrinsic PEEP, or prolonged expiratory time [29]. The spontaneous effort can cause greater local lung stress and overdistension by the negative inspiratory pleural pressure swing (diaphragm contraction), especially in dependent regions. It also results in substantial tidal recruitment, stretch, and then represents as air trapping [30, 31]. Previous studies have demonstrated the feasibility of capturing static regional air trapping with EIT [32, 33].

The occurrence of pendelluft may be attributed to alveolar heterogeneity or excessive spontaneous breathing effort, leading to gas movement within the lung and the higher risk of lung injury [34, 35]. That may explain the significance of the higher pendelluft incidence in the failure group. And due to the decrease in EELV during the weaning process, accompanied by the alveolar collapse, resulting in uneven gas distribution of the lung and then intensifying the pendelluft.

From a clinical standpoint, the absolute EELV_loss_ during the T-piece trial, rather than the PSV trial, carries the discriminative power for extubation failure. When using EELV_loss_ to assess the alveolar collapse during the weaning, it is necessary to take into account the possibility of air trapping.

### Gravity-dependent regional collapse

Dividing the lung into four ventro-dorsal EIT regions revealed that the additional volume loss in the failure group was localized predominantly to the dependent zones (ROI 3). In contrast, successful patients experienced a more homogeneous reduction when PEEP was withdrawn. This spatial divergence supports the concept that dorsal atelectasis precipitated by superimposed pressure is a key mechanism underlying T-piece intolerance. Interestingly, the same dorsal regions already displayed lower baseline ventilation in patients who later manifested the T-volume loss group. Thus, reduced dorsal recruitment at baseline may serve as an early warning sign that these patients will undergo critical derecruitment when exposed to the higher elastic load of the T-piece.

### *P*- and *T*-volume loss classification stratify risk beyond traditional indices

By integrating the time-course of EELV_loss_, we derived two distinct groups:

- P-volume loss (EELV_loss PSV_ > EELV_loss T-piece_) − volume loss front-loaded during the PSV phase, representing patients who tolerate volume loss early and stabilize thereafter; 74.4% of eventual successes fell into this category.
- T-volume loss (EELV_loss PSV_ < EELV_loss T-piece_) − volume loss accelerated during the T-piece phase, capturing 70.6% of failures.

The *T*-volume loss group was associated with worse dorsal ventilation at baseline, greater tidal-volume loss, higher respiratory rate, more pendelluft, and lower SpO_2_ during the T-piece trial. Importantly, neither traditional EIT-derived indices of inhomogeneity (GI, COV, RVD) nor bedside clinical variables distinguished success from failure as clearly as the classification based on EELV_loss_. This implies that real-time monitoring of regional EELV_loss_, rather than static snapshots of ventilation distribution, is required to forecast weaning behavior in high-risk cohorts.

### Clinical implications

Our data suggest that patients with poor dorsal ventilation at baseline who subsequently develop the T-volume loss pattern should be considered for alternative weaning strategies—e.g., extubation directly to non-invasive ventilation, prolonged low-level pressure support, or recruitment maneuvres—rather than proceeding to immediate extubation after a “successful” PSV-SBT. Conversely, the P-volume loss may identify a subset in whom PSV-SBT alone is sufficient, potentially shortening ventilation duration without increasing reintubation risk.

## Limitations

This current study has some limitations. First, we did not conduct randomization about SBT methods and perhaps cannot exclude selection bias; every patient received the two trials in the same order. Time-dependent phenomena such as progressive fatigue, accumulation of secretions, or prolongation of ventilator time could have amplified the EELV_loss_ observed during the T-piece phase and thus confounded our results. But PSV-SBT was always easier to pass, and we focused on the more challenging T-piece SBT in this study. Additionally, a crossover design was judged to be unsafe in this high-risk, post-cardiac surgery cohort because it would have obliged patients who had already failed a T-piece trial to be returned to PSV for a second, “late” PSV trial, prolonging total weaning time and possibly increasing the risk of cardiopulmonary deterioration. All 60 patients we enrolled in the analysis of the relationship of EELV_loss_ and T-piece SBT failure had the same duration weaning time. Second, as we selected PEEP 0 cmH_2_O as the beginning of PSV-SBT, EELV_loss_ at PSV-SBT 3 min reflected a combination of alveolar derecruitment due to PEEP removal and truly lung volume changes related to spontaneous breathing. These two effects cannot be separated in the current study design and might fundamentally affect the classification of “P-volume loss” versus “T-volume loss”. Third, as EELV_loss_ in the failure group was not significant during PSV-SBT, our study did not provide direct measurements of dynamic overinflation in this period; we just speculated the presence of air trapping according to the increased RR and decreased TIV, which may lead to EELV_loss_ that might not accurately reflect alveolar collapse or just apparent stability due to hyperinflation and compromise measurement validity. Third, we studied only patients deemed high-risk; extrapolation to low-risk populations requires caution. Finally, SBT duration was limited to 30 min per phase; longer trials might reveal additional volume loss or late pendelluft.

## Conclusion

In post-cardiac surgery patients at high risk of extubation failure, the transition from PSV-SBT to T-piece SBT is accompanied by a progressive, gravity-dependent EELV_loss_ that is more pronounced in those who ultimately fail. The simple EELV-based classification introduced here—*P*-volume loss and *T*-volume loss—provides a bedside tool to identify impending failure earlier than conventional indices, potentially guiding personalized weaning decisions and reducing unplanned reintubation.

## Supplementary Information

Supplementary Material 1

## Data Availability

The datasets used and/or analyzed during the current study are available from the corresponding author upon reasonable request.

## Abbreviations

EIT: Electrical impedance tomography
BMI: Body mass index
APACHE: Acute Physiology and Chronic Health Evaluation
EF: Ejection fraction
CABG: Coronary artery bypass grafting
CPB: Cardiopulmonary bypass
AOC: Aortic cross-clamping
P/F: Arterial partial pressure of oxygen to inspired fraction of oxygen ratio
HR: Heart rate
MAP: Mean arterial pressure
RR: Respiratory rate
NE: Norepinephrine
IMV: Invasive mechanical ventilation
VFD: Ventilator-free day
SBT: Spontaneous breathing trial
PSV: Pressure-support ventilation
EELI: End-expiratory lung impedance
EELV: End-expiratory lung volume
TIV: Tidal impedance variation
PEEP: Positive end-expiratory pressure
GI: Global inhomogeneity
COV: Center of ventilation
ROI: Regions of interest
RVD: Regional ventilation delay
ICU: Intensive care unit
SpO_2_: Peripheral oxygen saturation
IQR: Interquartile range
