# Supplementary material for: Effects of two ventilator-weaning methods on lung volume and ventilation distribution by electrical impedance tomography in post-cardiac surgery patients: a prospective cohort study
Source: J Intensive Care. 2026 Jan 23;14:17. doi: 10.1186/s40560-026-00850-1 (PMC12910891; doi:10.1186/s40560-026-00850-1)
Supplement: Supplementary file 1 — Supplementary Material 1 [file 40560_2026_850_MOESM1_ESM.docx]

Supplementary Table 1. Baseline characteristics and EIT parameters of all patients

| Variables | PSV-SBT success  N=60 | | PSV-SBT failure  N=7 | *P* |
| --- | --- | --- | --- | --- |
| Age, y | | 58±13 | 71±7 | **0.006** |
| Sex, M (%) | | 33 (55.0) | 3 (42.9) | 0.31 |
| BMI, Kg/m^2^ | | 24.1±3.2 | 24.8±3.1 | 0.53 |
| APACHE II Score | | 12±4 | 16±2 | **0.011** |
| Chronic respiratory disease, n(%) | | 11 (18.3) | 2 (28.6) | 0.17 |
| Pre-operative EF value | | 63 [55,67] | 64 [55,66] | 0.33 |
| Type of sugery | |  |  |  |
| CABG, n(%) | | 19 (31.7) | 3 (42.9) | 0.34 |
| Valve surgery, n(%) | | 20 (33.3) | 2 (28.6) | 0.58 |
| Others, n(%) | | 21 (35.0) | 2 (28.6) | 0.26 |
| Sugery process | |  |  |  |
| CPB time | | 133±38 | 155±30 | 0.12 |
| AOC time | | 89±33 | 117±12 | **0.003** |
| P/F, mmHg | | 339 [272, 416] | 321 [253, 406] | 0.58 |
| HR, bpm | | 90 [84, 96] | 87 [80,92] | 0.37 |
| MAP, mmHg | | 88 [79, 95] | 82 [72, 94] | 0.39 |
| RR, min^-1^ | | 15 [12, 18] | 18 [15, 20] | 0.12 |
| SpO_2_, % | | 99 [98, 100] | 98 [97, 100] | 0.21 |
| NE dose, ug/kg/min | | 0.01 [0.00, 0.12] | 0.08 [0.00, 0.17] | 0.46 |
| ROI 1+2 (%) | | 44 [53, 58] | 56 [53, 60] | 0.39 |
| ROI 3+4 (%) | | 45 [42, 47] | 44 [40, 47] | 0.39 |
| CoV | | 49.3 [47.6, 51.1] | 48.2 [46.7, 49.8] | 0.14 |
| GI | | 0.34 [0.32, 0.36] | 0.34 [0.33, 0.37] | 0.51 |
| Pendelluft (%) | | 11 (18.3) | 1 (14.3) | 0.15 |
| Extubation within 24h, n(%) | | 38 (63.3) | 0 (0.0) | **<0.001** |
| IMV days in total* | | 5 [4, 6] | 7 [6, 9] | **0.018** |
| VFDs at day 28 | | 23 [22, 24] | 21 [19, 22] | **0.013** |
| ICU stay, days | | 7 [6, 8] | 10 [8, 12] | **0.008** |
| Reintubation more than 24h, n(%) | | 0 (0.0) | 0 (0.0) | / |

BMI, body mass index; APACHE, acute physiology and chronic health evaluation; EF, ejection fraction; CABG, coronary artery bypass grafting; CPB, cardiopulmonary bypass; AOC, aortic Cross-Clamping; P/F, arterial partial pressure of oxygen to inspired fraction of oxygen ratio; HR, heart rate; MAP, mean arterial pressure; RR, respiratory rate; NE, Norepinephrine; SpO_2_, peripheral oxygen saturation; PSV, pressure support ventilation; SBT, spontaneous breathing trial; EIT, Electrical impedance tomography; GI, global inhomogeneity; COV, center of ventilation; ROI, regions of interest; RVD, regional ventilation delay; IMV, invasive mechanical ventilation; VFD, ventilator-free day; ICU, intensive care unit; *represents the period from the admission in ICU with mechanical ventilation to completely discontinuing mechanical ventilation, including the duration of reinitiating mechanical ventilation after SBT failure. Boldface indicates signiﬁcant P values.

Supplementary Table 2. Multivariate logistic regression analysis for T-piece SBT failure.

| Variables | B | S.E. | Wald | OR [95% CI] | *P* |
| --- | --- | --- | --- | --- | --- |
| T-volume loss | 2.287 | 0.834 | 7.519 | 9.847 [1.920, 50.494] | **0.006** |
| Age | 0.035 | 0.031 | 1.277 | 1.035 [0.975, 1.099] | 0.26 |
| APACHE II | 0.227 | 0.118 | 3.673 | 1.255 [0.995, 1.583] | 0.06 |
| CPB time | 0.033 | 0.020 | 2.674 | 1.034 [0.993, 1.076] | 0.10 |
| AOC time | -0.026 | 0.023 | 1.273 | 0.975 [0.933, 1.019] | 0.26 |

SBT, spontaneous breathing trial; APACHE, acute physiology and chronic health evaluation; CPB, cardiopulmonary bypass; AOC, aortic Cross-Clamping; OR, odds ratio; S.E. standard error; CI, Confidence Interval.

Supplementary Table 3. Multivariate logistic regression analysis for T-volume loss.

| Variables | B | S.E. | Wald | OR [95% CI] | *P* |
| --- | --- | --- | --- | --- | --- |
| Dorsal ventilation | -0.216 | 0.082 | 6.979 | 0.805 [0.686, 0.946] | **0.008** |
| Age | 0.056 | 0.033 | 2.924 | 1.058 [0.992, 1.128] | 0.09 |
| APACHE II | -0.065 | 0.092 | 0.510 | 0.937 [0.783, 1.121] | 0.48 |
| CPB time | 0.006 | 0.013 | 0.262 | 1.006 [0.982, 1.032] | 0.61 |
| AOC time | 0.004 | 0.013 | 0.105 | 1.004 [0.979, 1.030] | 0.75 |

APACHE, acute physiology and chronic health evaluation; CPB, cardiopulmonary bypass; AOC, aortic Cross-Clamping; OR, odds ratio; S.E. standard error; CI, Confidence Interval.

Supplementary Figure 1
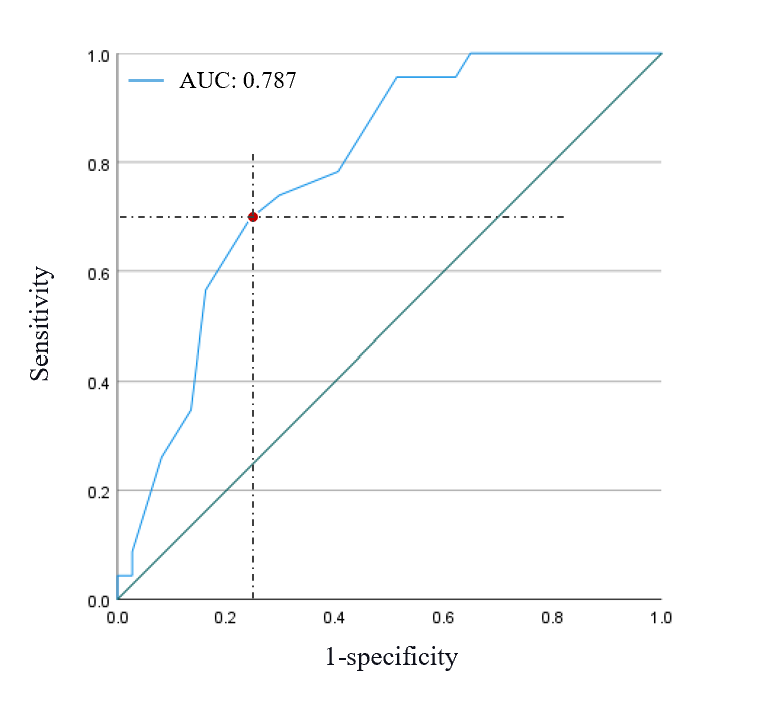
. ROC curve of baseline dorsal ventilation associated with T-volume loss.

ROC, receiver operating characteristic.
